# Cardiac Amyloid for the Internist

**DOI:** 10.7759/cureus.12915

**Published:** 2021-01-26

**Authors:** Hassan Ashraf, Adam Hafeez, Juan Vilaro

**Affiliations:** 1 Internal Medicine, University of Florida College of Medicine, Gainesville, USA; 2 Cardiology, University of Florida College of Medicine, Gainesville, USA

**Keywords:** cardiology, amyloid, internist, mri, echo, ekg, mps, light chain, dyscrasia

## Abstract

Cardiac amyloid is an uncommon cause of diastolic dysfunction the recognition of which requires the internist to have clinical suspicion to guide diagnosis and treatment. Cardiac amyloid is an infiltrative cardiomyopathy with significant morbidity and mortality. Appropriate diagnosis is important because management of cardiac amyloid differs from typical heart failure with preserved ejection fraction. An astute internist must be able to recognize common findings of cardiac amyloidosis. Here we present a case of a patient presenting with diastolic heart failure and the steps leading towards diagnosis and subsequent treatment.

## Introduction

Amyloidosis is a protein misfolding disorder causing severe morbidity and mortality depending on the affected organ. Cardiac amyloid carries the worst prognosis amongst the varying amyloidoses that affect other organ systems [1]. Cardiac amyloid previously thought to be an uncommon occurrence is now recognized as a common cause of difficult to treat diastolic heart failure [2]. Appropriate recognition based on clinical suspicion guides treatment. Here we present a case of a patient presenting with diastolic heart failure and being diagnosed and subsequently treated for cardiac amyloidosis.

## Case presentation

A 68-year-old female presented to ER with two days of chest tightness, dyspnea and leg edema. She had varicose vein ablation in a city six hours away three days prior to presentation and had been on steroids. She reported not feeling well since the drive back. She had no formal cardiac history but reported having a normal stress test nine years prior due to dyspnea. She was not on any cardiac medications and had never seen a cardiologist.

**Figure 1:**
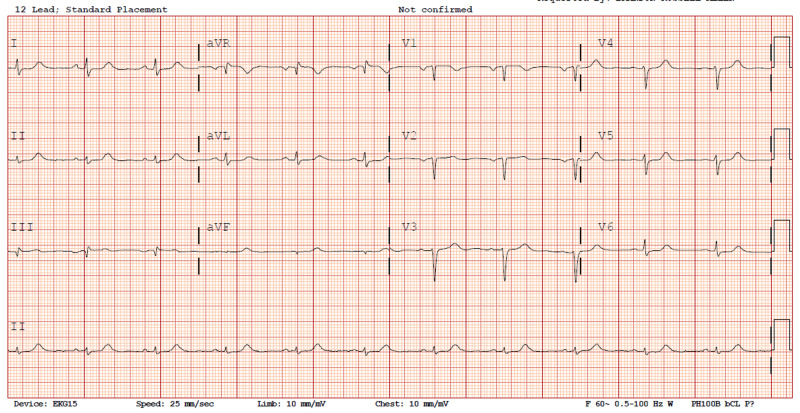
Electrocardiogram. The patient’s electrocardiogram displays feature low voltage in the limb leads (QRS amplitude less than 5mm in leads I, II, III, aVL, aVR, aVF) and pseudo infarct pattern (pathologic Q waves in V1, V2, V3, along with loss of R wave progression). The combination of low voltage on limb leads and pseudo infarct pattern have high specificity and positive predictive value for the diagnosis of cardiac amyloid [3].

**Figure 2:**
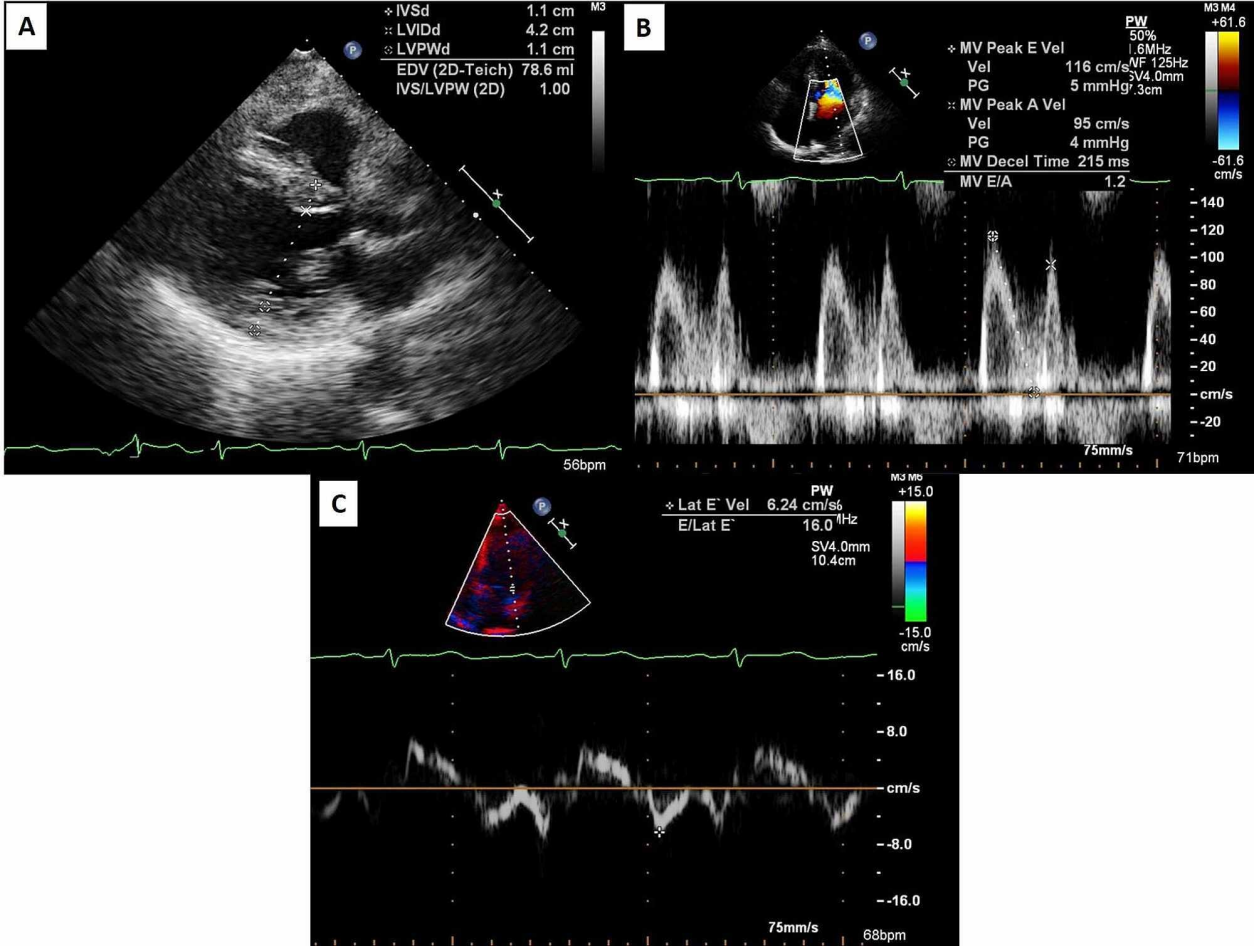
Echocardiogram. A:  Left ventricular wall thickness is demonstrated here using the parasternal long-axis view of the heart at end diastole. Septal thickness and posterior wall thickness are both measured at 1.1 cm which is higher than the normal range of 0.6-0.9 cm for both in females. B: Filling pressures are ascertained using doppler data gathered across the mitral valve in the apical view. The E and the A refer to two distinct waves created by the passive and active filling of the left ventricle respectively. Our patient's E/A ratio is 1.2 which is in the normal range of 0.75 and 1.5 making this a pseudonormal filling pattern. C: Measuring tissue doppler can provide the E/E' ratio which can help distinguish between normal and pseudonormal filling patterns. Our patient's E/E' shown here is 16 which is highly suggestive of elevated filling pressures. For reference, normal E/E' ratios are less than 8.

On admission, her evaluation was significant for mildly elevated BNP (118 pg/mL), normal troponins levels, elevated liver enzymes (AST/ALT: 70/116 IU/L), and a normal CT angiography of the chest. Electrocardiogram (EKG) showed low voltage QRS in all leads (Figure 1). She was given sublingual nitroglycerin and diuresis which improved her symptoms. A myocardial perfusion study (MPS) found a reversible perfusion defect in the mid to apical anterior wall. A bedside transthoracic echocardiogram (TTE) found mild left ventricular thickening with pseudo-normal left ventricle relaxation pattern and elevated left atrial pressures (Figure 2).

**Figure 3:**
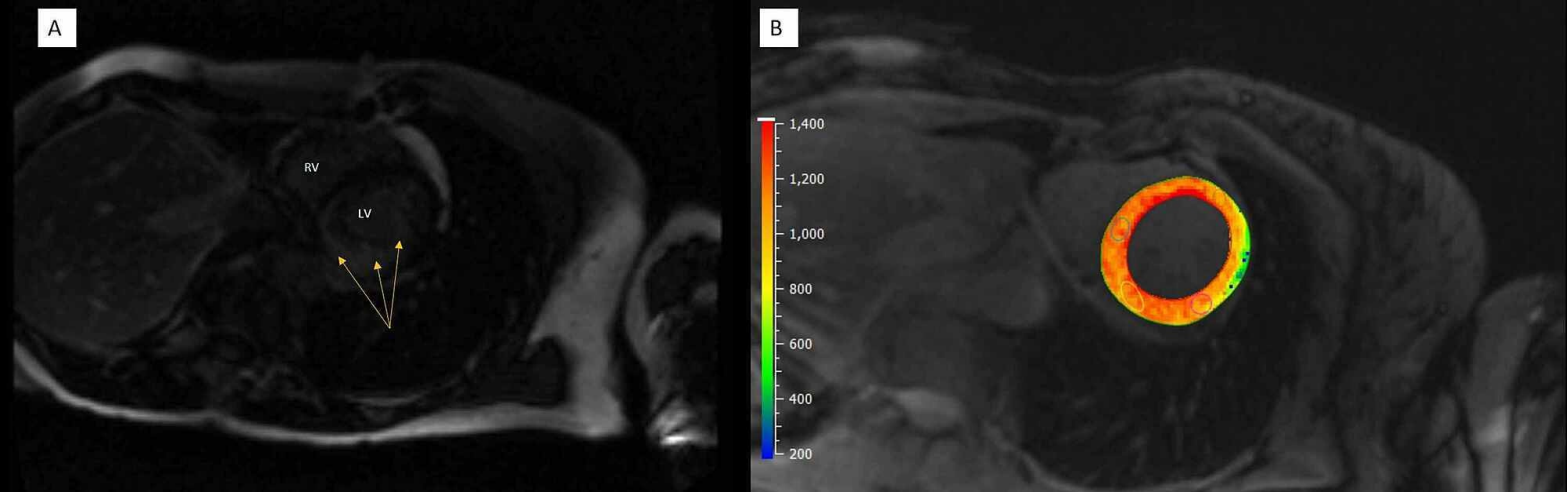
Cardiac MRI. A: Short axis view after gadolinium contrast administration. There is patchy delayed enhancement involving the mid-myocardial inferior wall. RV = right ventricle; LV = left ventricle. B: Short axis view T1 map showing significantly elevated T1 values highly suggestive of cardiac amyloid. Normal myocardium T1 value is 900 ms-1,000 ms on a 1.5T scanner.

Her presentation and constellation of findings on EKG and transthoracic echocardiogram raised concerns for cardiac amyloidosis. Further workup revealed elevated serum-free lambda levels to 12.45 mg/dL (normal: 0.57-2.63 mg/dL). Cardiac MRI demonstrated delayed gadolinium enhancement and T1 values suggestive of cardiac amyloid (Figure 3). 

Right heart catheterization with endomyocardial biopsy was performed and tissue stained with Congo red showed focal perivascular nodular aggregates of pink amorphous material suggestive of amyloid.

She underwent skeletal CT scan to search for lytic lesions to evaluate for multiple myeloma due to the elevated serum-free lamba levels. After the above workup was completed she was discharged with follow-up scheduled. On hematologic follow-up, she underwent bone marrow biopsy which was positive for amyloid deposition in small vessels and small number of abnormal plasma cells. Flow cytometry did not reveal monoclonal gammopathy and FISH testing was normal. Cardiac biopsy results revealed infiltrative AL (lambda)-type amyloid deposition. Her ultimate diagnosis is primary lambda chain amyloidosis with cardiac and bone marrow involvement. Her disease classification is Stage II for Mayo Clinic’s prognostication based upon her troponin and BNP. She is seen by oncology and started on CVD (cyclophosphamide, bortezomib, dexamethasone). She continued to follow with cardiology and oncology for treatment and monitoring.

## Discussion

There are over two dozen known protein types that cause amyloidosis and about one-third of them are known to have cardiovascular system involvement [4]. The two amyloidoses which most commonly affect the heart are immunoglobulin light chain (AL), and transthyretin related (TTR). AL amyloid cardiomyopathy carries a poor prognosis and is associated with the most morbidity and mortality. Untreated AL amyloid has a mean survival of six months from onset of heart failure. AL amyloid is male predominant occurring most commonly in the 5th and 6th decades of life. In contrast, TTR amyloid is slower progressing with survival in the range of years to decades [5].

AL and ATTR have many overlapping clinical features and share a similar tissue level pathogenesis with both toxic and infiltrative effects. The proteotoxicity from oxidative damage leads to cell death and fibrosis. In addition, the fibers impact the tissue structure causing stiffness and impaired contraction and relaxation as well as disturbed electrical conductance. The clinical presentation is directly related to the structural and toxic effects so patients present with heart failure, arrhythmias, and heart block. Systemic symptoms include neuropathy, autonomic dysfunction, and end-organ dysfunction [6]. Newer studies hint at increased rates of carpal tunnel, biceps tendon rupture, and lumbar spinal stenosis in patients with ATTR as opposed to AL [7].

Given the often plain presentation, internists have to be keen on the subtleties that separate cardiac amyloidosis from non-amyloid heart failure. AL Amyloidosis is a systemic disease with systemic manifestations. Muscular pseudohypertrophy, enlarged tongue, hepatomegaly, splenomegaly, neuropathy, autonomic dysfunction, increased bleeding, easy bruisability, and periorbital purpura are some examples of non-cardiac systemic findings in amyloidosis [8]. 

The EKG in cardiac amyloidosis will sometimes show a low voltage and/or pseudo-infarct pattern (pathologic Q waves in leads V1-V3). P waves can be abnormal in setting of atrial amyloid infiltration [9]. A TTE will be read by a cardiologist but an internist should be aware that cardiac amyloid will often cause concentric left ventricular (LV) hypertrophy with wall thickness often greater than 15mm which is unusual to see in other diseases such as hypertensive heart disease. In cardiac amyloidosis the heart will be echogenic, granular, and display a restrictive pattern along with diastolic dysfunction [10]. A cardiac MRI is a powerful tool which can be used to rule out non-amyloid causes of LV hypertrophy (LVH). Another imaging modality is a technetium pyrophosphate scan which can look specifically for ATTR amyloidosis.

Serum-free light-chain (FLC) assays combined with serum and urine immunofixation electrophoresis (IFE) have a sensitivity near 99% for AL amyloidosis [11] and offer an efficient and non-invasive way of screening patients suspected to have cardiac amyloid [12]. It is key to note that Serum protein electrophoresis (SPEP) is not enough to rule out AL-amyloid because unlike multiple myeloma (MM) the amount of circulating protein is a lot lower requiring the heightened sensitivity of a serum FLC assay. The serum FLC assay can also be used to monitor response to treatment.

The diagnosis of cardiac amyloidosis and specifically AL amyloidosis requires tissue sample with amyloid deposition and evidence of plasma cell dyscrasia. A tissue sample is obtained from an endomyocardial biopsy via heart catheterization. Plasma cell dyscrasia is demonstrated with a bone marrow biopsy [13].

Treatment for AL amyloid cardiomyopathy involves supportive care for the symptoms as well as treatment targeting the production of amyloid itself. Knowing how to provide supportive care is key for the internist. Heart failure symptoms are managed with diuretics, both loop diuretics and aldosterone antagonists. Unlike non-amyloid causes of heart failure, beta-blockers are often poorly tolerated. The stiff ventricles develop a restrictive pattern with a fixed stroke volume causing dependence on elevated heart rates to maintain cardiac output. Increased diastolic time does not increase end-diastolic volume [14]. The amyloid itself is treated with chemotherapy alone or in combination with stem cell transplant to target the plasma cell dyscrasia. Heart transplant is not frequently performed due to recurrence of amyloid within transplanted heart but there have been proposals for combined heart transplant along with chemotherapy and stem cell transplant [15].

## Conclusions

This case highlights a very common presentation of a patient who presented with dyspnea and chest pain. Patients with cardiac amyloidosis present similarly to other more routine cases of congestive heart failure not caused by an infiltrative cardiomyopathy. An EKG showing low voltage raised suspicion for an infiltrative process. TTE revealed a restrictive left ventricle pattern and a myocardial perfusion scan (MPS) showed reversible perfusion defect, though this region was not associated with a wall motion abnormality. Due to appropriate suspicion she had a further evaluation with serology and cardiac MRI which revealed an infiltrative process caused by amyloidosis. An astute internist with the right clinical suspicion can use the routine EKG, TTE, and MPS to further pursue workup of cardiac amyloid with serology and dedicated cardiac scans including MRI or technetium pyrophosphate scan with the help of a cardiology consultant. Patients who are incompletely diagnosed with heart failure with preserved ejection fraction or impaired diastolic function without further appropriate workup have little improvement in their symptoms and experience a rapid downward spiral in their health.
